# 3D-Printed Poly (l-lactic acid) Scaffolds for Bone Repair with Oriented Hierarchical Microcellular Foam Structure and Biocompatibility

**DOI:** 10.3390/biom15081075

**Published:** 2025-07-25

**Authors:** Cenyi Luo, Juan Xue, Qingyi Huang, Yuxiang Deng, Zhixin Zhao, Jiafeng Li, Xiaoyan Gao, Zhengqiu Li

**Affiliations:** 1 School of Material Science and Engineering, Xihua University, Chengdu 610039, China; luocenyi@163.com (C.L.); 0120230027@xhu.edu.cn (J.X.); 19981482996@163.com (Q.H.); 19881346165@163.com (Y.D.); zhixin_zhao@xhu.edu.cn (Z.Z.); 2 CCTEG Coal Mining Research Institute, Beijing 100013, China; ljfhngs@126.com; 3 Sichuan Institute for Drug Control (Sichuan Testing Center of Medical Devices, Sichuan Musk Deer Research Institute), Chengdu 611731, China

**Keywords:** poly (l-lactic acid), supercritical carbon dioxide, fused deposition modeling, continuous extrusion foaming, oriented hierarchical microporous, biocompatibility

## Abstract

This study proposes a continuous preparation strategy for poly (l-lactic acid) (PLLA) scaffolds with oriented hierarchical microporous structures for bone repair. A PLLA-oriented multi-stage microporous bone repair scaffold (hereafter referred to as the oriented multi-stage microporous scaffold) was designed using a novel extrusion foaming technology that integrates fused deposition modeling (FDM) 3D printing with supercritical carbon dioxide (SC-CO_2_) microcellular foaming technology. The influence of the 3D-printed structure on the microcellular morphology of the oriented multi-stage microporous scaffold was investigated and optimized. The combination of FDM and SC-CO_2_ foaming technology enables a continuous extrusion foaming process for preparing oriented multi-stage microporous scaffolds. The mechanical strength of the scaffold reached 15.27 MPa, meeting the requirements for bone repair in a low-load environment. Notably, the formation of open pores on the surface of the oriented multi-stage microporous scaffold positively affected cell proliferation, differentiation, and activity, as well as the expression of anti-inflammatory and pro-inflammatory factors. In vitro cell experiments (such as CCK-8) showed that the cell proliferation rate in the oriented multi-stage microporous scaffold reached 100–300% after many days of cultivation. This work provides a strategy for the design and manufacture of PLLA scaffolds with hierarchical microcellular structures and biocompatibility for bone repair.

## 1. Introduction

Poly (l-lactic acid) (PLLA) is a thermoplastic, aliphatic, semi-crystalline polyester known for its biocompatibility and biodegradability [1,2,3]. It is safely degraded through the same metabolic pathways as lactic acid [4]. Due to its non-irritant, biodegradable absorption, and ease of processing and molding characteristics, PLLA has been extensively utilized in biomedicine and tissue engineering. Over the past 30 years, PLLA has been safely employed in various clinical applications, including dissolvable sutures, intra-bone implants, and soft-tissue implants [4]. From a bionic perspective, preparing scaffold materials that closely mimic the structure of natural bone tissue can significantly enhance their biocompatibility. Although many researchers have explored the regulation of polylactic acid in bionic structures from different perspectives, studies on bone repair scaffold materials with multi-stage oriented micropores prepared through continuous extrusion foaming remain scarce.

Traditional methods for preparing porous PLLA include particulate leaching [5,6,7], supercritical carbon dioxide (SC-CO_2_) gas foaming [8,9,10,11], fused deposition modeling (FDM) [12,13,14], electrospinning [15,16,17,18,19], solvent casting [19,20], injection molding [21], freeze-fixation, freeze-gelation [22], and freeze-drying [7,23,24]. In addition to the SC-CO_2_ foaming method and FDM, most processes have the problem of organic solvent residue. Based on this, SC-CO_2_ foaming technology and FDM have become more environmentally friendly processes for the preparation of porous PLLA materials.

Among these, FDM offers advantages such as structural stability, personalization, minimal raw material usage, and high production efficiency. However, due to limitations in dimensional precision, the pore size of FDM-produced scaffolds typically ranges between 0.1–0.3 mm [25,26]. In contrast, the ideal pore size for bone repair materials ranges from 10–1000 μm. Studies have shown that scaffolds with apertures of 60–1200 μm can effectively stimulate cell proliferation and differentiation [27,28]. Furthermore, pore distribution, shape, and porosity are critical factors that influence bone tissue growth [29]. FDM technology alone is insufficient for precise control of the structure of bone repair scaffolds.

The supercritical carbon dioxide foaming method utilizes a non-toxic, non-flammable, and cost-effective supercritical fluid to prepare porous structures. This simple approach avoids the use of residual pore-forming agents associated with traditional foaming methods, thereby reducing potential risks to the human body. Additionally, it offers advantages in optimizing cell size stability and processing conditions, such as reduced pressure requirements [9]. The integration of SC-CO_2_ with FDM printing technology addresses the limitations of 3D printing in pore structure fabrication. This combination enhances the preparation of bone repair materials, making them more diverse, structurally comprehensive, and closer to natural bone. It also mitigates the structural and functional limitations of using a single technology.

However, the combination of SC-CO_2_ foaming and FDM printing is often limited to intermittent processing, where post-printing foaming can cause scaffold deformation. This study proposes a novel strategy for the design and manufacture of PLLA scaffolds for bone repair with hierarchical microcellular structures and biocompatibility. The low-temperature solid-phase nucleation of CO_2_ in the matrix in SC-CO_2_ microcellular foaming technology was combined with the high-temperature melt extrusion process of FDM. In the process of FDM deposition printing, a continuous extrusion foaming process is formed with the expansion and merger of the pre-constructed gas nucleus. A continuous extrusion foaming process is applied to the filaments, producing scaffolds with improved structural integrity and functionality.

## 2. Experimental Section

### 2.1. Materials

PLLA was purchased from Nature Works Co., Nebraska, USA, with the commercial trade number 3052D. The d-lactic acid content is 4%. The melt index (210 °C, 2.16 kg), glass temperature (T_g_), melting temperature (T_m_), and density of PLLA are listed in Table 1. Commercial-purity grade CO_2_ (99% purity, Air Liquide) was used as the solid-phase nucleation agent.

### 2.2. Preparation of Samples

#### 2.2.1. Preparation of FDM Filaments

The filaments required for FDM printing were prepared using a single-screw extruder (Nanjing Sheng chi Rubber Machinery Manufacturing Co., Ltd., SHJ-35, Nanjing, China), and the diameter of the extruded filaments was controlled at 1.5 ± 0.2 mm to ensure the size requirements of 3D printing. Before extruding the filaments, the raw material PLLA was dried at 80 °C for 5 h to ensure that the water content of the raw material was less than 0.025% (250 ppm). During extrusion, the plasticizing temperature was maintained at 175 °C, and the extruder head was maintained at 140 °C to ensure the formation of filaments. The winding speed controlled the diameter of the extruded filaments.

#### 2.2.2. Preparation of Solid Phase Nucleation Filaments

**Figure 1:**
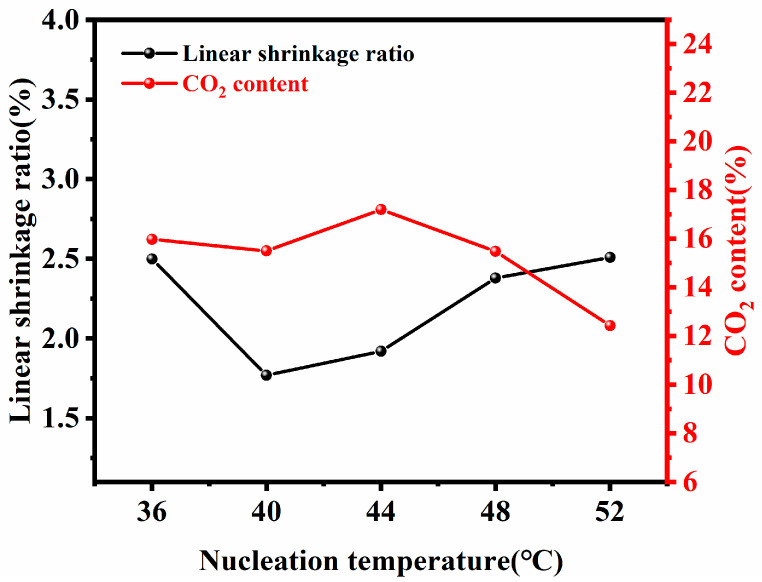
Comparative analysis of the linear shrinkage and CO_2_ content of filaments obtained by nucleation at different temperatures.

The extruded PLLA filaments were placed in an SC-CO_2_ foaming apparatus (SZWEICO, Beijing, China) and maintained at 40 °C and 10 MPa for 40 min. The nucleation process conditions were determined based on Figure 1. The analysis indicated that a nucleation temperature of 40 °C minimized the linear shrinkage of the substrate filaments. Although the content of CO_2_ is moderate at this temperature, considering that the linear shrinkage will affect the size of the substrate filament, it is not conducive to the size stability required for subsequent FDM printing consumables. Therefore, 40 °C was selected as the optimal nucleation temperature. During this process, the SC-CO_2_ fluid dissolves into the PLLA filaments and nucleates within the internal solid phase. The pressure was then released at a rate of 1 MPa/min. Once the internal pressure equilibrated with the atmospheric pressure, the nucleated PLLA filaments were removed and allowed to cool at room temperature.

#### 2.2.3. Preparation of Oriented Multi-Stage Microporous Scaffold by Extrusion Foaming Method

**Figure 2:**
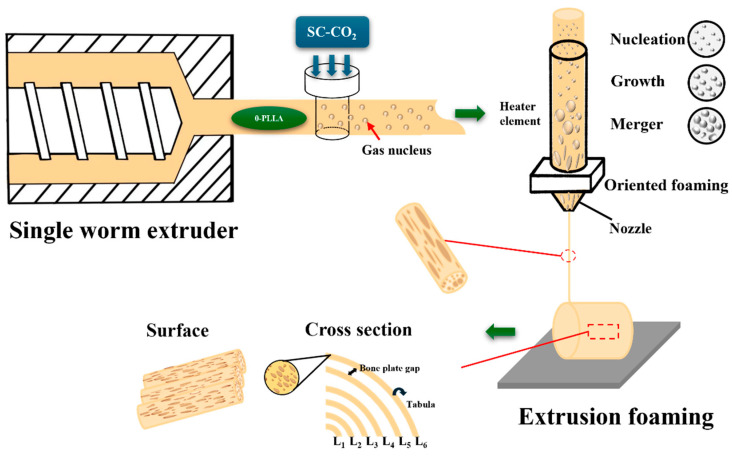
Structure construction and core idea diagram.

**Figure 3:**
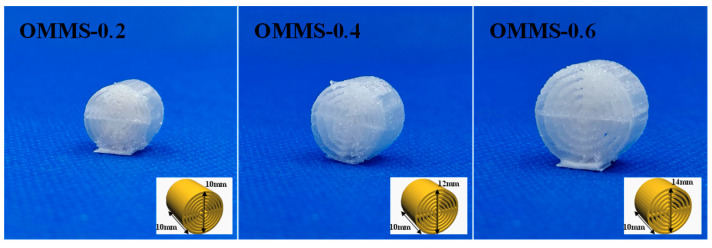
Images of the 3D-printed PLLA scaffold and the macropore size distribution graph.

PLLA filaments with SC-CO_2_ solid-phase nucleation were used for FDM printing (Goofoo Tech (Xiamen). Co., Ltd., Nova, Xiamen, China) consumables. The scaffold design was inspired by the Harvard bone plate of a natural load-bearing bone as a reference to establish a concentric cylindrical scaffold model with a bionic structure. The structural design and core concepts are illustrated in Figure 2. Among them, L_1_–L_6_ are respectively named as the arrangement order of bone plates inside the scaffold from inside to outside. Considering that the glass transition temperature of PLLA ranges from 50–66 °C, the nozzle temperature was set to 190 °C and the platform temperature to 50 °C to ensure a stable 3D printing process. This setup provided sufficient bonding force and prevented the sample from collapsing or deforming due to softening. The detailed printing parameters are shown in Table 2. The spacing between each layer of the concentric cylinder was uniform, and adjustments to this spacing were used to regulate the bone-plate gap. Oriented multi-stage microporous scaffolds with different bone plate gaps (0.2 mm, 0.4 mm, and 0.6 mm) prepared by extrusion foaming were named OMMS-0.2, OMMS-0.4, and OMMS-0.6, respectively. The non-foamed PLLA bone repair scaffold prepared by FDM is referred to as the NFS. The PLLA plate is named PLLA-P. The single-layer bone plate is referred to by the name of the scaffold and the number of layers. For example, the second layer of the oriented multi-stage microporous scaffold with a 0.2 mm bone plate gap is referred to as OMMS-0.2-L_2_. The scaffold prepared is shown in Figure 3.

### 2.3. Measurements

#### 2.3.1. Differential Scanning Calorimetry (DSC)

The melting behavior of the PLLA samples in a nitrogen atmosphere was characterized using a DSC instrument (DSC Q200, TA Instruments, New Castle, USA).

##### Non-Isothermal Crystallization and Melting Behavior

A 5-10 mg sample was placed in a crucible and equilibrated at 40° C for 5 min under a nitrogen (N_2_) atmosphere. Subsequently, the temperature was increased to 200 °C at a rate of 10 °C/min, and the heat flow curve was recorded. The crystallinity (X_c_) of the sample was calculated using Formula (1):

(1) $$X_{c}=\left(\frac{∆H_{m}-∆H_{c}}{{∆H}_{m}^{0}}\right)\times 100\%$$

where $∆H_{m}$ represents the melting enthalpy change during the test, $∆H_{c}$ denotes the enthalpy change during the cold crystallization process of the sample, and $∆H_{m}^{0}$ is the melting enthalpy of fully crystallized PLLA, with a value of 93.6 J/g [30].

#### 2.3.2. X-Ray Diffractometer (XRD)

The XRD test was performed at room temperature using an X-ray diffractometer (DX-2500, Dandong Aolong Ray Instrument Group Co., Ltd., Liaoning, China). A copper target (CuKa, λ = 0.154 nm) was used to scan the range of 10–40° at a scan rate of 0.03/s and an accelerated current of 20 mA.

The microcrystalline size of the material was calculated using Scherrer’s Formula (2):

(2) $$L_{khl}=\frac{K\lambda }{\beta cos\theta }$$

where *L_khl_* represents the grain size (nm) perpendicular to the lattice plane, λ is the wavelength of the incident X-ray (nm), θ is the Bragg diffraction angle, and K is the crystal shape factor, which depends on the shape of the microcrystalline and the lattice plane coefficient, β and *L_khl_*. When β_1/2_ is defined as the full width at half-maximum of the peak, the value of K is typically taken as 0.9.

#### 2.3.3. Scanning Electron Microscopy (SEM)

A scanning electron microscope (TESCAN MIRA3, Czech Republic, China, Shanghai) was used to examine the sample morphology at an operating voltage of 5 kV. The morphology, pore structure, and dispersion of the samples were observed using scanning electron microscopy [31].

#### 2.3.4. Mechanical Property Measurement

A single-layer-oriented multi-stage microporous scaffold was used to test the mechanical properties. The practical application effect of the scaffold in the in vitro body fluid simulation environment was evaluated over a simulation cycle of 0–7 days. The samples were immersed in a PBST solution (mass volume ratio of the sample to the solution was 1:30) and sealed in a dual-function water bath thermostatic oscillator (SHA-B, Changzhou Yitong Analytical Instrument Manufacturing Co., Ltd.), maintaining low-speed oscillation conditions at 37 °C. Three groups of parallel samples were taken on days 0, 1, 3, and 7 to test the tensile strength. The mechanical properties of the samples were tested using a type 4302 material testing machine (Instron, USA) at a speed of 5 mm/min. The uniaxial test was repeated three times at room temperature, and the results were averaged. The tensile strength was calculated using Formula (3).

(3) $$\sigma =\frac{F}{S}$$

where *σ* stands for tensile strength in MPa, F is the axial tension (the maximum stress when it is broken), with a unit of N, and S is the cross section (original cross-sectional area) of the material specimen with a unit of mm^2^.

#### 2.3.5. Biological Evaluation

In this study, immortalized *mouse* bone marrow-derived macrophages (iBMDM) and *mouse* embryonic fibroblasts (NIH-3T3) were used to evaluate the biocompatibility of the oriented multi-stage microporous scaffold. Before the experiment, the cells were passed 2-3 times. The cells were cultured in Dulbecco’s Modified Eagle Medium (DMEM; Gibco, USA) supplemented with 10% fetal bovine serum (FBS; Gibco) and 1% penicillin-streptomycin-gentamicin (PSG; Solarbio). Before cell inoculation, the samples were pretreated with 75% ethanol and sterilized under ultraviolet light for 1 h.

##### Cell Viability and Proliferation

The sample was placed in a well and digested with trypsin (Gibco), and the resulting cell suspension was collected by centrifugation. The cells were seeded onto polystyrene (TCPS) plates at a density of 2.5 × 10^4^ cells per well and incubated at 37 °C with 5% carbon dioxide and 95% humidity for 1, 3, and 7 days. The medium was replaced every other day. After incubation, the samples were removed, and 200 µL of a pre-prepared live dye solution (calcein-AM/PI) was added to each well and incubated for 30 min. The solution was discarded, and the cells were washed with PBS buffer. Images were captured for analysis using a Nikon Eclipse Ti2 (Nikon, Japan) inverted fluorescence microscope.

##### Cell Morphology

A scanning electron microscope (SEM, TESCAN MIRA3, Czech Republic) was used to observe the morphology of the samples after co-culturing with iBMDM and NIT-3T3. Before observation, the samples were rinsed three times with PBS, fixed in 2.5% glutaraldehyde for 3 h, dehydrated through a gradient ethanol series (40%, 60%, 80%, and 100%, *v*/*v*), and vacuum-dried at −50 °C overnight. The SEM operating conditions were consistent with those used for surface morphology observations.

##### Gene Expression Analysis

iBMDM were seeded onto the material at a density of 10^5^ cells/well for co-culture. After 3 and 7 days, total RNA was extracted from the implanted cells using TRIzol (Vazyme, Nanjing, China) and reverse-transcribed into cDNA using Hiscript II Reverse Transcriptase (Vazyme, Nanjing, China). The expression levels of pro-inflammatory-related genes (*IL-1β*, *IL-6*, and *TNF-α*) and anti-inflammatory-related genes (*IL-10* and *TGF-β*) were determined using quantitative fluorescence PCR.

#### 2.3.6. Surface Tension Measurement

The contact angles of the control and oriented multi-stage microporous scaffold samples were measured and recorded using a contact angle measuring instrument (JH-901A, China Jinhuayi (Beijing) Technology Co., Ltd., Beijing, China). Water and ethylene glycol (EG) were used as the test media, with a droplet size of 15 µL. Each material group was measured three times, and the results were averaged. The contact angles of water on the PLLA plate (WCA-0) and oriented multi-stage microporous scaffold monolayer bone plate (WCA-1), and EG on the PLLA plate (EGCA-0) and scaffold monolayer bone plate (EGCA-1) were tested. The surface tension was measured using the Owens-Wendt-Raeble-Kaeble (OWRK) regression model. The following formulas were applied:

(4) $$\frac{1+cos\theta }{2}\frac{\gamma_{l}}{\sqrt{\gamma_{l}^{d}}}=\sqrt{\gamma_{s}^{d}}+\sqrt{\gamma_{s}^{p}}\sqrt{\frac{\gamma_{l}^{p}}{\gamma_{l}^{d}}}$$

(5) $$\gamma_{s}=\gamma_{s}^{p}+\gamma_{s}^{d}$$

(6) $$\gamma_{s-soybean\;oil}={\left[\sqrt{\gamma_{soybean\;oil}^{p}}-\sqrt{\gamma_{s}^{p}}\right]}^{2}+{\left[\sqrt{\gamma_{soybean\;oil}^{d}}-\sqrt{\gamma_{s}^{d}}\right]}^{2}$$

where θ represents the contact angle of the test liquid, and $\lambda_{l}$ denotes the surface energy of the liquid. The parameters $\gamma_{l}^{p}$ and $\gamma_{l}^{d}$ refer to the Polar component and Dispersion components of the test liquid, respectively.

## 3. Results and Discussion

The abbreviations corresponding to each group of samples are shown in Table 3.

### 3.1. Thermal Properties and Crystal Structure

#### 3.1.1. DSC

**Figure 4:**
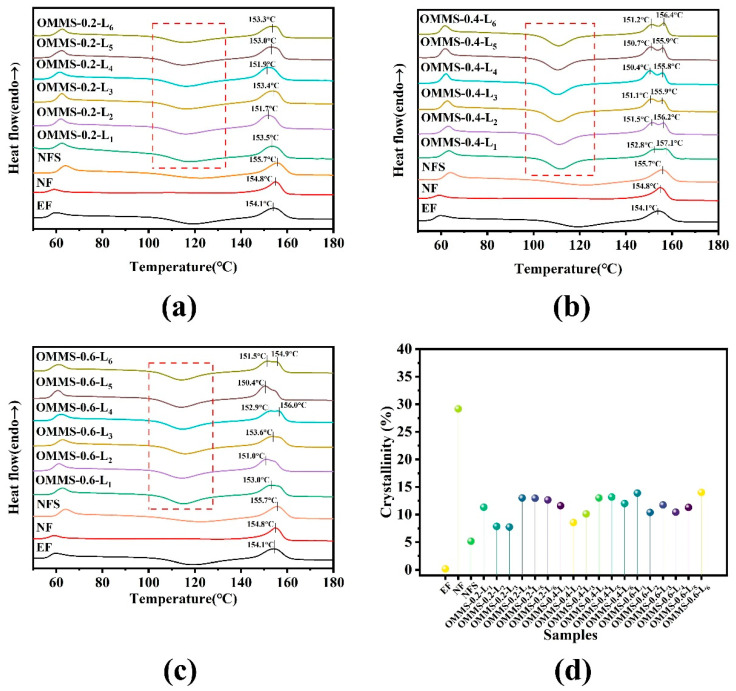
DSC non-isothermal crystallization test diagram of different samples. The melting behaviors (**a**–**c**) and crystallinities (**d**) of the different samples were compared.

The DSC test results for the samples are shown in Figure 4. Extruded filament (EF), SC-CO_2_ solid-phase nucleation filament (NF), and non-foamed PLLA scaffold (NFS) were compared with oriented multi-stage microporous scaffolds.

By comparing the non-isothermal DSC curves of each group of samples, the cold crystallization peak appeared in the extruded filament, indicating that there were defects in the internal crystal structure. After FDM printing, T_g_ and T_m_ increased, and the cold crystallization peak decreased. The high-temperature melting process in the FDM printing process broke the original crystal structure, and the molecular chain was rearranged. Combined with the stress field of printing stretching, the local molecular chain was oriented, and the rapid cooling of the filament reduced the fluidity of the molecular chain. After the solid-phase nucleation of the extruded filament, the T_g_ changed slightly, the T_m_ increased, and the cold crystallization peak disappeared. During the solid-phase nucleation process of SC-CO_2_, the CO_2_ fluid acted as a plasticizer, enhanced the movement ability of the molecular chain inside the fiber, induced the crystallization of PLLA, and formed a relatively complete crystallization zone. During heating at a faster heating rate, the presence of a crystalline region limits the movement of the molecular chain, preventing the appearance of an obvious cold crystallization peak. However, in the DSC non-isothermal crystallization curve of each layer of the oriented multi-stage microporous scaffold prepared by further extrusion foaming, the T_g_ increased but was lower than that of the non-foamed scaffold. In the process of extrusion foaming, a tensile stress field exists, and the molecular chain orientation, local ordering, and crystal structure are improved; however, at the same time, foaming interference occurs, which reduces the compactness and order of the internal orientation structure. In addition, cold crystallization peaks reappear after extrusion foaming. In the melting stage of the extruded foam filaments, the perfect crystallization area was destroyed at a higher temperature, forming a partial crystalline state, and the internal crystalline structure of the oriented multi-stage microporous scaffold was defective. The calculation and analysis of the crystallinity in Figure 4d also reflect this phenomenon. After low-temperature solid-phase nucleation, the crystallinity inside the nucleation filament was significantly higher than that of the extruded filament, increasing from 0.18% to 29.17%. After extrusion foaming, the crystallinity of the substrate with low-temperature solid-phase nucleation decreased to 10%. Although the high-temperature melting process of extrusion foaming interferes with the perfection of the crystalline structure, the induced crystallization of SC-CO_2_ and the induced orientation of extrusion foaming still greatly promote the structural perfection of the scaffold. The crystallinity of the oriented multi-stage microporous scaffold was still higher than that of the non-foamed PLLA scaffold.

The DCS results of the oriented multi-stage microporous scaffolds showed a melting double-peak phenomenon, in which OMMS-0.4 exhibited a significantly stable melting double peak. The temperature difference between the high- and low-temperature peaks was 5 °C, and the high-temperature T_m_ was the highest among the components. It is speculated that during the extrusion foaming process, the orientation combination during the foaming and printing processes forms a high-temperature and high-stress field, which promotes the formation of a relatively disordered β crystal phase with a disordered molecular chain arrangement. OMMS-0.4 exhibited the sharpest cold crystallization peak and fast and uniform crystallization behavior. Combined with the heat dissipation space and cooling rate brought by the suitable gap structure, it promotes the formation of a highly ordered crystal structure and forms the α crystal phase. The actual crystal form and transformation, combined with the XRD test results, were analyzed as follows.

#### 3.1.2. XRD

**Figure 5:**
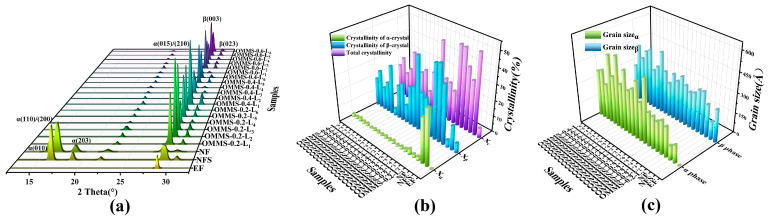
XRD test results (**a**) and statistical comparison of crystalline (**b**) and grain size (**c**) of oriented multi-stage microporous scaffolds with different bone plate gaps.

The XRD test results and statistical analysis of the crystallinity and grain size of the samples are shown in Figure 5.

In Figure 5a, the extruded substrate filament only exhibits a weak β (003) [32,33,34] diffraction peak induced by the high-temperature tensile orientation stress field. The diffraction peaks of α (010), α (110)/(200), α (203), and α (015)/(210) [34,35,36,37,38,39] appeared after solid-phase nucleation or FDM printing. This indicates that the extruded filament formed a complete and ordered α-crystal phase after SC-CO_2_ plasticization-induced crystallization. The high-temperature orientation stress field induced by FDM printing locally ordered the molecular chain and induced crystallization. From the comparative analysis of the morphological distribution of the peak in Figure 5a and the crystallinity in Figure 5b, the crystallinity of the extruded substrate filament was low, and the crystallinity could be significantly improved by FDM printing or solid-phase nucleation. The cold crystallization peak of the DSC non-isothermal crystallization curve (Figure 4) weakens and disappears owing to the improvement in the crystalline structure. As shown in Figure 5a,b, the high-temperature orientation of FDM printing promoted the formation of α crystals (α (110)/(200), α (203), α (015)/(210)) and β crystals (β (003), β (023)) [33,34]. The plasticization of solid nucleation is more conducive to the formation of α crystals (α (010), α (110)/(200), α (203), α (015)/(210)), and the diffraction peak corresponding to the β crystal (β (003), β (023)) is weak.

In addition, the crystalline structure and crystalline phase of the oriented mult-istage microporous scaffold prepared by the extrusion foaming process were analyzed. It was found that, except for a small amount of α crystal (α (015)/(210)) retention, the high-temperature orientation and the high-stress field caused by foaming greatly increased the β crystal (β (003)) in the scaffold. Based on the change in crystallinity and the shape of the diffraction peak, it can be inferred that there is a crystal transformation from the α crystal to the β crystal, which is consistent with the analysis of the crystal structure change by DSC test results. Figure 5b,c show that the oriented multi-stage microporous scaffold has high crystallinity and large grain size, and the crystal structure is mainly contributed by the β crystal. This indicates that the high-temperature and high-stress field environment generated by the extrusion foaming process enhances the mobility of the molecular chain, which is conducive to overcoming the lattice energy barrier to realize molecular migration and rearrangement. The molecular chain has sufficient conditional motion orientation; the regularity of the crystal structure is improved, the structure is more perfect, and it is conducive to the formation of β crystals and induces the transformation of α crystals into β crystals.

### 3.2. Morphology and Mechanical Properties

#### 3.2.1. Morphology and Structure

**Figure 6:**
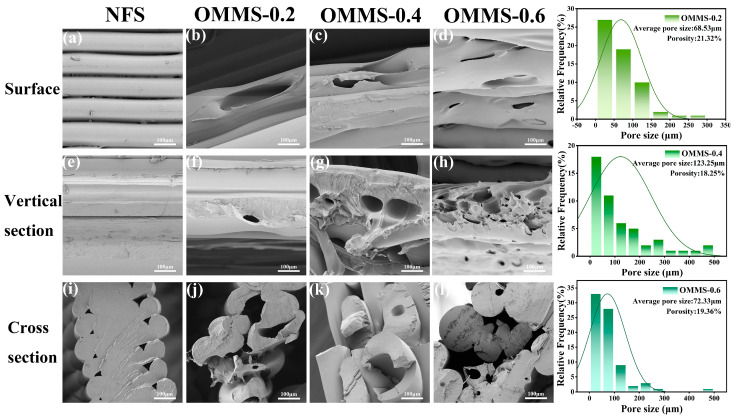
SEM images (100 μm), pore size distribution, and porosity analysis of different samples.

The structures and morphologies of the samples were analyzed using SEM, and the resulting images are presented in Figure 6.

As shown in Figure 6a,e,i, the non-foamed PLLA scaffold exhibited a macroscopic orientation structure of the substrate. In addition, there was no adhesion between the bone plates of each layer. In addition to the gully structure formed between the filaments, the surface and longitudinal section of the substrate were smooth. In the cross-section, neat and uniform triangular (or fan-shaped) holes were observed. Combined with the analysis of the transverse and longitudinal sections, penetrating holes with a diameter of 30–50 μm were observed in the scaffold. The oriented multi-stage microporous scaffold retains the macro-oriented structure of the substrate, while the internal and external structures of the scaffold differ in size.

During the extrusion foaming process, the foaming process was combined with the stretching orientation of FDM printing, and many ellipsoidal bubbles (2–500 μm) were formed inside the scaffold, along with a gully structure formed by partially broken bubbles. Combined with the analysis of the pore size distribution and porosity of oriented multi-level microporous scaffolds with different bone plate gaps, the porosity of the scaffolds prepared by the extrusion foaming process is about 20%, and the pore size is mainly distributed in the range of 60–130 μm. Among them, OMMS-0.6 has the most uniform pore structure, and the oriented multi-stage microporous scaffolds with different bone plate gaps all have distinct oriented multi-stage microporous structures.

In addition, the bone plates in each gradient sample were not adhered to, and the gap between the bone plates did not disappear, with large through holes (200, 400, and 600 μm). In the oriented multi-stage microporous scaffold, due to the influence of the extrusion foaming process, the size of the small through-holes formed by the deposition between the substrates changed but still existed.

By analyzing the structure of natural load-bearing bone, it was found that the size of intraosseous micropores was mainly distributed in three levels of 10–30 μm (accommodating osteocytes), 100–500 μm (new tissues such as blood vessels and nerves grow into space), and 200–500 μm (nutrient transport and bone metabolite excretion), forming a multi-level micropore structure [40].

In addition, combined with the concentric lamellar orientation structure of the bone plate, the oriented multi-level microporous structure in the natural bone meets the basic physiological functions and sports load-bearing requirements. By analyzing the SEM results, it was found that the macro-oriented structure and multi-level microporous structure of the oriented multi-stage microporous scaffolds prepared by the extrusion foaming process had a certain degree of agreement with the structure of natural load-bearing bone, and the bionic concept was introduced into the construction of the oriented multi-stage microporous scaffold structure. In summary, the oriented multistage microporous scaffolds produced by the extrusion foaming process meet the structural and functional requirements of bone repair scaffolds.

#### 3.2.2. Mechanical Property

The strength of the single-layer structure of the oriented multi-stage microporous scaffold was tested using a universal testing machine. The test results show that the tensile strength of the oriented multi-stage microporous scaffold prepared by the extrusion foaming process reached 15.27 MPa. In an in vitro body fluid simulation environment, the tensile strength of the scaffold decreased from 14.94 MPa to 12.98 MPa and 12.31 MPa on days 0, 3, and 7, respectively. After 3 days, the mechanical properties tended to be stable, and the mechanical properties only lost 8.65% on the 7th day, showing good stability. The SEM image in Figure 6 shows an oriented multi-level microporous structure inside the scaffold, and the macroscopic orientation of the substrate and the local orientation of the molecular chain enhance the strength of the material. The multi-level stress dispersion adjustment of multi-stage micropores is coordinated with the complex bearing environment of the body. Natural long bone is composed of two different bone structures: cancellous bone and cortical bone. According to the literature, the mechanical strength of cancellous bone is 4–12 MPa, while the mechanical strength of cortical bone is 130–180 MPa [41]. The mechanical strength of the oriented multi-stage microporous scaffolds in this study exceeded the strength requirements of cancellous bone and has been able met the repair needs of some small bone defects or small fractures, especially in smaller weight-bearing sites, such as fingers, toes, or non-load-bearing sites in elderly patients.

### 3.3. Biological Properties

#### 3.3.1. Biocompatibility

**Figure 7:**
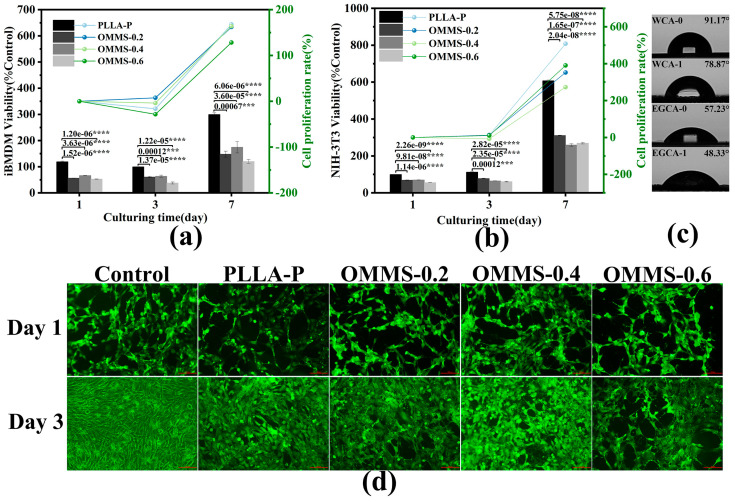
Biocompatibility characterization of the samples. Proliferation of iBMDM (**a**) and NIH-3T3 (**b**), contact angle test (**c**), and cell staining experiment of NIH-3T3 (100 μm) (**d**). (*** *p* < 0.001, **** *p* < 0.0001).

The biocompatibility of the PLLA plate (hot-pressed PLLA smooth plate with the same processing temperature) and the oriented multi-stage microporous scaffold prepared by extrusion foaming were characterized, as shown in Figure 7, including the proliferation experiments of two types of cells (iBMDM and NIH-3T3) in different samples, live/dead staining experiments of NIH-3T3 cells, and contact angle test. The surface energy of the sample was summarized, and the biocompatibility of the material was analyzed in combination with its structural characteristics.

As shown in Figure 7a,b,d, the cells were inoculated into different components of the sample for several days, and the cells proliferated well. After 3 days of cultivation, the cell proliferation rate on the oriented multi-stage microporous scaffold was generally higher than that on the PLLA plate. As shown in Figure 7a, the cell proliferation rate on the PLLA plate decreased after 3 days of cultivation. After 7 days of culture, cell proliferation in all components was significantly improved. The cell proliferation rate of iBMDM was more than 100% on the 7th day, and the cell proliferation rate of NIH-3T3 was more than 250% after 7 days of culture, indicating that the oriented multi-stage microporous scaffold had low biological toxicity and good biocompatibility [42,43]. It can be found that after 3 days of culture, the cell proliferation in the oriented multi-stage microporous scaffold is generally better than that on the PLLA plate, and after 7 days of culture, the cell proliferation on the scaffold is the same as that on the PLLA plate, and the cell proliferation rate of most components is lower than that of the PLLA plate. According to Figure 7c, the surface energy parameters of the corresponding samples were calculated using the contact angle test for water and ethylene glycol, as shown in Table 4.

The contact angle on the surface of the oriented multi-stage microporous scaffolds prepared by the extrusion foaming method decreased, indicating that the hydrophilicity of the extruded foam samples was enhanced. The surface energy of the scaffold was 34.96 mN/m, whereas that of the PLLA plate in the control group was 36.96 mN/m. The wettability and hydrophilicity of the scaffold increased significantly. A higher solid surface energy is beneficial for wetting, which proves that the wettability and hydrophilicity of the oriented multi-stage microporous scaffold surface prepared by extrusion foaming are enhanced. This improvement is attributed to the surface characteristics of the scaffold, which not only has a gully structure formed by the parallel arrangement of the substrate filaments but also produces multi-stage micropores during the foaming process. The combination of gully and pore structures increases the surface roughness and further improves the wettability and hydrophilicity of the scaffold. Therefore, the surface effect promotes cell adhesion and stimulates cell differentiation, thereby increasing the cell proliferation rate.

Combined with Figure 7a,b,d, it was found that the OMMS-0.4 showed a good ability to promote cell proliferation for both cell types. Among the oriented multi-stage microporous scaffolds with different bone plate gaps, the scaffolds prepared using the extrusion foaming method exhibited similar internal porous structures and consistent pore sizes. Therefore, the bone plate gap is a significant influencing factor. Compared with OMMS-0.4, OMMS-0.2 and OMMS-0.6 provided a less effective cell-contact surface. When the gap between the bone plates is too small, cell contact is limited to the surface, making it difficult for cells to penetrate the grooves and gully structures. In contrast, when the gap is too large, the internal contact increases, but the interlayer interaction effect is missing. A 0.4 mm bone plate gap achieves an optimal balance, so that the entire cell is simultaneously subjected to structural deepening and interlayer interaction in the gap, thereby stably promoting cell proliferation and ensuring that the material has excellent biocompatibility.

After 7 days of culture, each component showed significant cell proliferation. At the same time, the cell proliferation rate of the PLLA plate was significantly improved, even better than that of some oriented multi-stage microporous scaffold components. Although the surface of the PLLA plate was smooth and lacked structural stimulation, the open surface promoted the absorption and excretion of nutrients and metabolites by cells adhered to the surface and provided sufficient space for cell proliferation. This cell proliferation only acts on the surface of the PLLA material, and the bone repair process is complex and long, including the needs of various physiological functions, involving the growth of new tissues and the coordination of material structure. It cannot be determined whether the good biocompatibility of the PLLA plate in the short term is the optimal solution for bone repair materials.

#### 3.3.2. Cell Morphology

**Figure 8:**
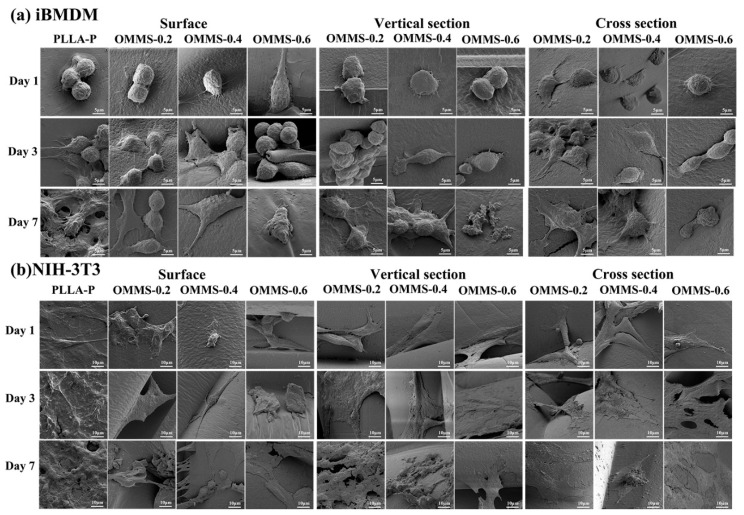
Growth morphology of iBMDM (**a**) and NIH-3T3 (**b**) after 1, 3, and 7 days of inoculation in different samples.

The growth morphology of the two cell types on different samples after 1, 3, and 7 days of inoculation is shown in Figure 8. As shown in Figure 8a, the growth of iBMDM gradually improved over time in all sample groups. Taking OMMS-0.4 as an example, the cells on the first day showed fewer antennae, low cell activity, and limited growth. After 3 days of culture, the cell antennae increased, the cells spread, tended to differentiate, and adhesion to the matrix was enhanced. Finally, after 7 days of culture, the cells differentiated and closely connected, and the cell mass spread outward to form a biofilm. Cells attached and proliferated on each side of the scaffold, and cell activity was good. In addition, the cell activity of iBMDM inoculated with OMMS-0.6 tended to decrease on the 3rd day. After 7 days of culture, the cells showed no obvious cell antennae, cell death, failure to form biofilms, or poor cell activity.

The growth of NIH-3T3 on different components (Figure 8b) also improved with increasing culture time, and a spreading cell membrane was formed after 7 days of culture. The growth of NIH-3T3 cells seeded on OMMS-0.4 was used as an example. In the early stages of culture, cell activity was good, the antennae were obvious, and there was a trend of connection and differentiation between the cells. Over time, cells further differentiated and spread, eventually forming a highly diffuse biofilm. At the same time, the growth of NIH-3T3 in the scaffold was better than that on the surface of the scaffold, and there was better cell activity in the oriented multi-stage microporous scaffold with a larger bone plate gap.

From the growth morphology of the two types of cells on differently oriented multi-stage microporous scaffolds, it can be analyzed that the scaffold prepared by the extrusion foaming method has the characteristics of stimulating cell activation, proliferation, and differentiation. The combination of the macroscopic orientation of the scaffold substrate and the broken pores constitutes a gully structure, and the multi-level hierarchical microporous structure increases the surface roughness of the oriented multi-stage microporous scaffold. The combination of these two provides a surface effect that promotes cell adhesion and activation.

At the same time, based on cell morphology and differentiation, the single surface effects of each bone plate are interrelated and combined in the bone plate gap to further promote cell activation and differentiation, so that the cells in the oriented multi-stage microporous scaffold have better activity than the cells on the surface of the scaffold. The volume and characteristics of the cells themselves also affect the promoting effect of the scaffold on cell activation and differentiation. From Figure 8, the size of NIH-3T3 is about 30 μm, and the size of iBMDM is distributed in 10–15 μm. NIH-3T3 forms a more relaxed and broad cell membrane. Combined with cell characteristics, both cell types adapted to a near-neutral environment, but iBMDM had narrower adaptability to the pH of the environment. During cell cultivation on oriented multi-stage microporous scaffolds, PLLA was hydrolyzed to produce lactic acid, which made the environment acidic. It is speculated that this is the reason why NIH-3T3 cells have better activity. In addition, according to cell characteristics, the proliferation ability of iBMDM is relatively low. NIH-3T3 has stronger proliferation ability and adaptability under different culture conditions, can grow and expand rapidly, and can continue to grow under high-density conditions. In oriented multi-stage microporous scaffolds, cell growth space is limited, the environment is complex, and the oriented multi-level microporous structure has a comprehensive effect, which is speculated to be another reason for the better activity of NIH-3T3. In summary, the oriented multi-stage microporous scaffold prepared by the extrusion foaming process had a positive effect on cell growth and morphological evolution.

#### 3.3.3. Gene Expression Analysis

**Figure 9:**
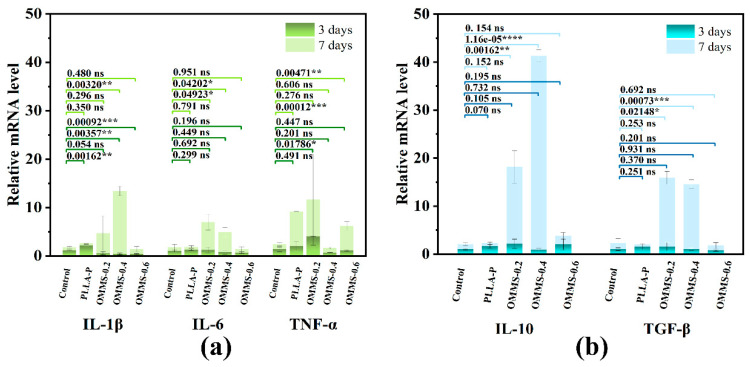
Expression of pro-inflammatory (**a**) and anti-inflammatory factors (**b**) in different components (ns, *p* > 0.05; * *p* < 0.05,** *p* < 0.01,*** *p* < 0.001,**** *p* < 0.0001).

During bone repair, the inflammatory response directly impacts the success and effectiveness of the process. Biomolecules that promote or enhance the inflammatory response are classified as pro-inflammatory factors, while those that reduce or terminate the inflammatory response are referred to as anti-inflammatory factors. The dynamic balance between pro-inflammatory and anti-inflammatory cytokines determines the development and outcome of inflammation. Pro-inflammatory cytokines activate the body’s innate and acquired immune systems during pathogen invasion, helping to eliminate the invaders. Conversely, anti-inflammatory cytokines work to resolve inflammation after the invaders are eliminated, restoring the body to normal immune and physiological states. Existing studies have shown that the shape, chemical, and physical properties of the implant material may cause changes in the intensity and duration of the inflammatory process, in which the surface properties and material composition of the material directly affect the immune response [44,45]. To evaluate this balance, iBMDM were inoculated in each sample group, and the expression levels of pro-inflammatory (*IL-1β*, *IL-6*, *TNF-α*) and anti-inflammatory factors (*TL-10*, *TGF-β*) were measured. The results are shown in Figure 9.

Compared to the control and PLLA plate groups, the cells inoculated on the oriented multi-stage microporous scaffold with different bone plate gaps exhibited increased expression of both pro-inflammatory and anti-inflammatory cytokines after 7 days of culture. This indicates that the oriented multi-stage microporous scaffold developed in this study effectively promoted the expression of cytokines in iBMDM. Among the scaffolds, OMMS-0.2 and OMMS-0.4 showed the most significant promotion of both pro-inflammatory and anti-inflammatory factors. This is because the physical properties of the material have an important influence on cell behavior and the expression of active factors.

The oriented micropores and gully structures on the surface of the scaffold give the material a higher surface roughness and surface effects, which can provide more attachment sites and make it easier for cells to adhere, thus promoting cell activation and proliferation. The cells present a more complex morphology, which enhances the interaction and signal transmission between cells and promotes the expression of inflammatory factors. Notably, the scaffolds enhanced the expression of anti-inflammatory factors compared to that of pro-inflammatory factors. This phenomenon was particularly significant for OMMS-0.4. This is critical because the normal occurrence and development of inflammation require a balance of cytokines, and minimizing the impact of inflammation is essential for effective bone repair. A treatment approach that slightly favors anti-inflammatory effects while maintaining the overall cytokine balance is more conducive to bone repair. In summary, OMMS-0.4 is a more suitable structure for the expression of immune factors. In summary, the oriented multi-stage microporous scaffold prepared by the extrusion foaming method has an excellent promoting effect on the expression of cellular immune factors. Among them, the scaffold with a 0.4 mm bone plate gap structure was found to be more suitable for the expression of immune factors.

### 3.4. Technology Optimization and Prospects

In this study, a continuous extrusion foaming process combining SC-CO_2_ foaming technology and FDM was initially implemented to prepare oriented multi-stage microporous PLLA bone repair scaffolds. Through various performance tests and characterizations, the current work shows that the continuous preparation process of extrusion foaming has better positive feedback and provides a new entry point for subsequent technical optimization. Phenomena such as polymorphism (α and β crystal phases) and melting double peaks (150–157 °C) have inspired the next step of research and provided theoretical support for subsequent research.

## 4. Conclusions

It is feasible to continuously prepare PLLA-oriented multi-level microporous bone repair scaffolds using the extrusion foaming method. During the extrusion foaming process, SC-CO_2_ plasticized and induced crystallization, and the high-stress field formed by the combination of high-temperature tensile orientation and released gas formed bubbles, optimizing the melting crystallization behavior, significantly improving the crystallinity inside the scaffold, and improving the crystal structure. The scaffold has an oriented multi-stage microporous structure (pore size of 2–500 μm and bone plate gap of 200–600 μm) that fits the natural load-bearing bone structure [34]. The mechanical strength of the oriented multi-stage microporous scaffold was 15.27 MPa, and the mechanical strength loss was less than 10% after 7 days in a simulated body fluid environment in vitro. It has good stability and can theoretically meet the needs of bone repair in a low-load environment [46]. The cell proliferation rate of iBMDM was more than 100% on the 7th day, and the cell proliferation rate of NIH-3T3 cells was more than 250% after 7 days of culture. The oriented multi-level microporous scaffold has good biocompatibility, which can stimulate cell adhesion, cell activation, proliferation, and differentiation, provide cells with good morphology and activity, promote the balanced regulation and release of cellular immune factors, and promote biological function [42,43,47,48]. It was also found that the oriented multi-stage microporous scaffold with a bone plate gap of 0.4 mm had advantages in promoting cell proliferation and differentiation and the expression of pro-inflammatory and anti-inflammatory factors.

## Figures and Tables

**Table 1:** Characteristics of the PLLA used.

Materials	Melt Index (g/10 min)	T_g_ (°C)	T_m_ (°C)	Density (g/cm^3^)
PLLA	14	50~66	145~160	1.24

**Table 2:** Parameters utilized for 3D printing.

Temperature (°C)		Speed (mm/s)	Adhesion
Nozzle	Platform		
190	50	60	Brim

**Table 3:** Sample abbreviation name comparison table.

Abbreviation	Samples	Remark
EF	Extruded filament	-\-
NF	SC-CO_2_ solid-phase nucleation filament	-\-
NFS	Non-foamed PLLA scaffold	-\-
OMMS	Oriented multi-stage microporous scaffold	L_1–6_ represents the number of bone plate layers.
OMMS-0.2	Oriented multi-stage microporous scaffold with a bone plate gap of 0.2 mm	L_1–6_ represents the number of bone plate layers.
OMMS-0.4	Oriented multi-stage microporous scaffold with a bone plate gap of 0.4 mm	L_1–6_ represents the number of bone plate layers.
OMMS-0.6	Oriented multi-stage microporous scaffold with a bone plate gap of 0.6 mm	L_1–6_ represents the number of bone plate layers.
PLLA-P	PLLA plate	-\-
WCA	Contact angles of water	0 refers to PLLA plate, 1 refers to scaffold monolayer bone plate
EGCA	Contact angles of EG	0 refers to PLLA plate, 1 refers to scaffold monolayer bone plate

**Table 4:** Surface energy parameters of the samples.

Samples	Contact Angle (°)		γ_s_^d^	γ_s_^p^	γ_s_
	Water	EG	(mN/m)	(mN/m)	(mN/m)
PLLA-P	91.17	57.23	35.79	1.17	36.96
OMMS	78.87	48.33	28.14	6.82	34.96

## Data Availability

Supporting data for the study results are available from the corresponding author upon reasonable request.

## References

[1] Zhang S., Shi X., Miao Z., Zhang H., Zhao X., Wang K., Jian Q., Guang Z. (2022). 3D-Printed Polyurethane Tissue-Engineering Scaffold with Hierarchical Microcellular Foam Structure and Antibacterial Properties. Adv. Eng. Mater..

[2] Ju J., Peng X., Huang K., Li L., Liu X., Chitrakar C., Chang L., Gu Z., Kuang T. (2019). High-performance porous PLLA-based scaffolds for bone tissue engineering: Preparation, characterization, and in vitro and in vivo evaluation. Polymer.

[3] Cao Y., Su J., Xiao Y., Ren J., Algadi H., Yeszhanov E., Sartayeva A., Huang J., Zhan G., Tynybekov B. (2025). Functional biomass/biological macromolecular phase change composites and their applications in different scenarios: A review. Int. J. Biol. Macromol..

[4] Fitzgerald R., Bass L.M., Goldberg D.J., Graivier M.H., Lorenc Z.P. (2018). Physiochemical characteristics of poly-L-lactic acid (PLLA). Aesthetic Surg. J..

[5] Lim J.I., Park H.K. (2012). Fabrication of macroporous chitosan/poly (l-lactide) hybrid scaffolds by sodium acetate particulate-leaching method. J. Porous Mater..

[6] Ezzati P., Ghasemi I., Karrabi M., Azizi H., Fortelny I. (2014). Preparation of porous PLLA/PCL blend by a combination of PEO phase and NaCl particulate leaching in PLLA/PCL/PEO/NaCl blend. Iran. Polym. J..

[7] Dong Y.S., Guo C., Lin P.H., Yin L.H., Pu Y.P. (2005). Preparation of porous poly (L-lactic acid) (PLLA) scaffold by porogen leaching and freeze drying. Key Eng. Mater..

[8] Dong S., Wang L., Li Q., Chen X., Liu S., Zhou Y. (2017). Poly (L-lactide)-grafted bioglass/poly (lactide-co-glycolide) scaffolds with supercritical CO_2_ foaming reprocessing for bone tissue engineering. Chem. Res. Chin. Univ..

[9] Athanasoulia I.G., Louli V., Schinas P., Rinotas V., Douni E., Tarantili P., Magoulas K. (2022). The effect of foaming process with supercritical CO_2_ on the morphology and properties of 3D porous polylactic acid scaffolds. Polym. Eng. Sci..

[10] Xu L.Q., Huang H.X. (2014). Foaming of poly (lactic acid) using supercritical carbon dioxide as foaming agent: Influence of crystallinity and spherulite size on cell structure and expansion ratio. Ind. Eng. Chem. Res..

[11] Chen J., Yang L., Chen D., Wang M., Wu L. (2023). Facile fabrication of highly interconnected poly (lactic acid) -based scaffolds with good hydrophilicity using supercritical carbon dioxide. J. Appl. Polym. Sci..

[12] Rajzer I., Kurowska A., Jabłoński A., Jatteau S., Śliwka M., Ziąbka M., Menaszek E. (2018). Layered gelatin/PLLA scaffolds fabricated by electrospinning and 3D printing-for nasal cartilages and subchondral bone reconstruction. Mater. Des..

[13] Yen H.J., Tseng C.S., Hsu S., Tsai C.S. (2009). Evaluation of chondrocyte growth in the highly porous scaffolds made by fused deposition manufacturing (FDM) filled with type II collagen. Biomed. Microdevices.

[14] Bhagia S., Bornani K., Agrawal R., Satlewal A., Ďurkovič J., Lagaňa R., Bhagia M., Yoo C., Zhao X., Kunc V. (2021). Critical review of FDM 3D printing of PLA biocomposites filled with biomass resources, characterization, biodegradability, upcycling and opportunities for biorefineries. Appl. Mater. Today.

[15] Yang H., Wang L., Xiang C., Li L. (2018). Electrospun porous PLLA and poly (LLA-co-CL) fibers by phase separation. New J. Chem..

[16] Qi Z., Yu H., Chen Y., Zhu M. (2009). Highly porous fibers prepared by electrospinning a ternary system of nonsolvent/solvent/poly (l-lactic acid). Mater. Lett..

[17] Yu Q.Z., Qin Y.M. (2013). Fabrication and formation mechanism of poly (L-lactic acid) ultrafine multi-porous hollow fiber by electrospinning. Express Polym. Lett..

[18] Muniyandi P., Palaninathan V., Veeranarayanan S., Ukai T., Maekawa T., Hanajiri T., Mohamed M.S. (2020). ECM mimetic electrospun porous poly (l-lactic acid) (PLLA) scaffolds as potential substrates for cardiac tissue engineering. Polymers.

[19] Vaquette C., Frochot C., Rahouadj R., Wang X. (2008). An innovative method to obtain porous PLLA scaffolds with highly spherical and interconnected pores. J. Biomed. Mater. Res. Part B: Appl. Biomater. Off. J. Soc. Biomater. Jpn. Soc. Biomater. Aust. Soc. Biomater. Korean Soc. Biomater..

[20] Selvam S., Chang W.V., Nakamura T., Samant D.M., Thomas P.B., Trousdale M.D., Mircheff A.K., Schechter J.E., Yiu S.C. (2009). Microporous poly (L-lactic acid) membranes fabricated by polyethylene glycol solvent-cast/particulate leaching technique. Tissue Eng. Part C Methods.

[21] Ghosh S., Viana J.C., Reis R.L., Mano J.F. (2008). Development of porous lamellar poly (l-lactic acid) scaffolds by conventional injection molding process. Acta Biomater..

[22] Ho M.H., Kuo P.Y., Hsieh H.J., Hsien T.Y., Hou L.T., Lai J.Y., Wang D.M. (2004). Preparation of porous scaffolds by using freeze-extraction and freeze-gelation methods. Biomaterials.

[23] Sasaki T., Tanaka K., Morino D., Sakurai K. (2011). Morphology and Release Kinetics of Protein-Loaded Porous Poly (L-Lactic Acid) Spheres Prepared by Freeze-Drying Technique. Int. Sch. Res. Not..

[24] Kim J.W., Taki K., Nagamine S., Ohshima M. (2008). Preparation of poly (L-lactic acid) honeycomb monolith structure by unidirectional freezing and freeze-drying. Chem. Eng. Sci..

[25] Buj-Corral I., Bagheri A., Petit-Rojo O. (2018). 3D printing of porous scaffolds with controlled porosity and pore size values. Materials.

[26] Winarso R., Anggoro P.W., Ismail R., Jamari J., Bayuseno A.P. (2022). Application of fused deposition modeling (FDM) on bone scaffold manufacturing process: A review. Heliyon.

[27] Prananingrum W., Naito Y., Galli S., Bae J., Sekine K., Hamada K., Tomotake Y., Wennerberg A., Jimbo R., Ichikawa T. (2016). Bone ingrowth of various porous titanium scaffolds produced by a moldless and space holder technique: An in vivo study in rabbits. Biomed. Mater..

[28] Li G., Wang L., Pan W., Yang F., Jiang W., Wu X., Kong X., Dai K., Hao Y. (2016). In vitro and in vivo study of additive manufactured porous Ti6Al4V scaffolds for repairing bone defects. Sci. Rep..

[29] Liang H., Chao L., Xie D., Yang Y., Shi J., Zhang Y., Xue B., Shen L., Tian Z., Li L. (2022). Trabecular-like Ti–6Al–4V scaffold for bone repair: A diversified mechanical stimulation environment for bone regeneration. Compos. Part B Eng..

[30] Södergård A., Stolt M. (2002). Properties of lactic acid based polymers and their correlation with composition. Prog. Polym. Sci..

[31] Huang J., Zeng X., Yu W., Zhang H., Min Y. (2025). Polyimide-based porous carbon/Ni nano-particle composites prepared by phase separation method with broadband absorption characteristics. Diam. Relat. Mater..

[32] Takahashi K., Sawai D., Yokoyama T., Kanamoto T., Hyon S.H. (2004). Crystal transformation from the α-to the β-form upon tensile drawing of poly (l-lactic acid). Polymer.

[33] Sawai D., Takahashi K., Imamura T., Nakamura K., Kanamoto T., Hyon S.H. (2002). Preparation of oriented β-form poly (l-lactic acid) by solid-state extrusion. J. Polym. Sci. Part B Polym. Phys..

[34] Li Y., Zhao Z., Huang Q., Luo C., Chen W., Gao X., Wang K., Li Z., Liu L. (2024). Preparation and properties of polydimethylsiloxane-regulated oriented microporous poly (L-lactic acid) biomimetic bone repair materials. Int. J. Biol. Macromol..

[35] Zhang J., Duan Y., Sato H., Tsuji H., Noda I., Yan S., Ozaki Y. (2005). Crystal modifications and thermal behavior of poly (L-lactic acid) revealed by infrared spectroscopy. Macromolecules.

[36] Di Lorenzo M.L., Cocca M., Malinconico M. (2011). Crystal polymorphism of poly (l-lactic acid) and its influence on thermal properties. Thermochim. Acta.

[37] Sarasua J.R., Prud’Homme R.E., Wisniewski M., Borgne A.L., Spassky N. (1998). Crystallization and melting behavior of polylactides. Macromolecules.

[38] Zhang J., Tashiro K., Tsuji H., Domb A.J. (2008). Disorder-to-order phase transition and multiple melting behavior of poly (L-lactide) investigated by simultaneous measurements of WAXD and DSC. Macromolecules.

[39] Cocca M., Di Lorenzo M.L., Malinconico M., Frezza V. (2011). Influence of crystal polymorphism on mechanical and barrier properties of poly (l-lactic acid). Eur. Polym. J..

[40] Jia Z., Xu X., Zhu D., Zheng Y. (2023). Design, printing, and engineering of regenerative biomaterials for personalized bone healthcare. Prog. Mater. Sci..

[41] Feng P., Jia J., Liu M., Peng S., Zhao Z., Shuai C. (2021). Degradation mechanisms and acceleration strategies of poly (lactic acid) scaffold for bone regeneration. Mater. Des..

[42] Zhang B., Wang L., Song P., Pei X., Sun H., Wu L., Zhou C., Wang K., Fan F. (2021). 3D printed bone tissue regenerative PLA/HA scaffolds with comprehensive performance optimizations. Mater. Des..

[43] Huang Y., Mao Y., Li H., Wang E., Mai H., Zhang W., Wen J., You H., Long Y., Guo W. (2025). 3D-Printed Thermally Activated Shape Memory PLA/TBC Composite Scaffold with Body-Compatible Temperature for Minimally Invasive Bone Repair. ACS Appl. Polym. Mater..

[44] Raghavendran H.R.B., Natarajan E., Mohan S., Krishnamurithy C., Murali M.R., Parasuraman S., Singh S., Kamarul T. (2021). The functionalization of the electrospun PLLA fibrous scaffolds reduces the hydrogen peroxide induced cytokines secretion in vitro. Mater. Today Commun..

[45] Soares D.G., Zhang Z., Mohamed F., Eyster T.W., Costa C.A.S., Ma P.X. (2018). Simvastatin and nanofibrous poly (l-lactic acid) scaffolds to promote the odontogenic potential of dental pulp cells in an inflammatory environment. Acta Biomater..

[46] Gao C., Gao J., You X., Huo S., Li X., Zhang Y., Zhang W. (2005). Fabrication of calcium sulfate/PLLA composite for bone repair. J. Biomed. Mater. Res. Part A Off. J. Soc. Biomater. Jpn. Soc. Biomater. Aust. Soc. Biomater. Korean Soc. Biomater..

[47] Li Y., Feng Y., Zhao Z., Liu L., Li Z., Li J. (2025). Biomimetic 2D-oriented microporous PLLA scaffolds: Fabrication and evaluation for bone repair. Int. J. Biol. Macromol..

[48] Yao H., Wang J., Deng Y., Li Z., Wei J. (2023). Osteogenic and antibacterial PLLA membrane for bone tissue engineering. Int. J. Biol. Macromol..

